# Time- and Cost-Efficient, Minimally Invasive Comparative Assessment of Implant Stability: Reliability and Inter-Examiner Agreement of IST Versus ISQ Across Different Bone Quality Models

**DOI:** 10.3390/bioengineering13010086

**Published:** 2026-01-12

**Authors:** Sung-Joon Kim, Se Hoon Kahm

**Affiliations:** 1 Department of Dentistry, Jeju National University Hospital, College of Medicine, Jeju National University, Jeju 63241, Republic of Korea; samuelsj@jejunu.ac.kr; 2 Department of Dentistry, Eunpyeong St. Mary’s Hospital, College of Medicine, The Catholic University of Korea, Seoul 03312, Republic of Korea

**Keywords:** implant stability, IST, ISQ, reliability, inter-examiner agreement, bone quality, dental implants

## Abstract

This study evaluated the reliability and inter-examiner agreement of the Implant Stability Test (IST) by Anycheck compared to the established Implant Stability Quotient (ISQ) by Osstell across different bone quality types. Seven dental hygienists with varying experience levels performed stability measurements using both devices on standardized implant models representing hard, normal, and soft bone qualities. Both IST and ISQ demonstrated excellent inter-examiner reliability (ICC > 0.90) across all bone quality types, with strong positive correlations (r > 0.85) between measurements regardless of bone density. No significant differences were found in measurement consistency between examiners with different experience levels for either device. The results demonstrate that IST provides comparable reliability to ISQ for implant stability assessment, with excellent inter-examiner agreement and accessibility for practitioners with varying experience levels. The IST system offers practical advantages including elimination of SmartPeg requirements, reduced abutment manipulation, and simplified measurement protocols, supporting its potential as a reliable and cost-effective alternative to traditional ISQ measurements under standardized experimental conditions.

## 1. Introduction

Implant stability assessment plays a fundamental role in determining the success of dental implant treatment, influencing decisions regarding loading protocols and long-term prognosis [1]. The evaluation of implant stability has evolved from subjective clinical assessments to objective, non-invasive measurement techniques that provide quantifiable data for clinical decision-making [2].

Resonance frequency analysis (RFA), particularly through the Osstell ISQ (Integration Diagnostics AB, Gothenburg, Sweden) system, has emerged as the most widely accepted method for implant stability assessment [3]. The ISQ system measures the resonant frequency of the implant–bone complex using a SmartPeg transducer, providing values on a scale of 0–100 that correlate with implant stability [4]. Despite its widespread acceptance and proven reliability, the ISQ system requires specific SmartPeg attachments and has associated costs that may limit its accessibility in some clinical settings [5]. In this context, alternative non-invasive techniques such as acoustic modal analysis (AMA) and percussion-based methods, including Periotest, have been explored as potential adjuncts to resonance frequency analysis, with previous studies reporting significant correlations with ISQ values [6,7].

Recent developments in implant stability measurement have introduced alternative approaches, including damping and percussion-based methods. The Anycheck IST (Implant Stability Test, Neobiotech Co., Ltd., Seoul, Republic of Korea) represents one such innovation, utilizing a different measurement principle that does not require SmartPeg attachments [8]. This device measures energy dissipation following a controlled impact, providing stability measurements that may offer advantages in terms of ease of use and clinical accessibility [9].

Previous studies have demonstrated strong correlations between IST and ISQ measurements. Lee et al. [4] reported a robust positive correlation (r = 0.981, *p* < 0.01) between ISQ and IST values, while demonstrating that healing abutment diameter had no statistically significant effect on IST measurements. Lee et al. [10] showed that both RFA and DCA methods displayed similar trends in primary implant stability measurement across different bone densities and implant lengths, with the Anycheck device offering greater ease of use compared to traditional RFA systems.

The influence of bone quality on implant stability measurements represents another critical consideration. Both measurement methods must effectively differentiate between different bone quality types while maintaining consistent reliability profiles [10,11]. In-ter-examiner reliability is a fundamental requirement for any clinical measurement tool, particularly considering the need for accessibility to practitioners with varying experience levels.

The primary objective of this study was to evaluate the reliability and inter-examiner agreement of the Anycheck IST compared to the Osstell ISQ across different bone quality types. Secondary objectives included assessing the correlation between the two measurement methods and evaluating the influence of examiner experience on measurement consistency.

## 2. Materials and Methods

### 2.1. Study Design

This was a controlled laboratory study designed to compare the reliability and inter-examiner agreement of two implant stability measurement devices: the Anycheck IST and the Osstell ISQ. The study protocol was designed to simulate clinical measurement conditions while maintaining standardized experimental parameters.

### 2.2. Bone Quality Models

Three standardized bone quality models were fabricated to represent different clinical bone types commonly encountered in implant dentistry:

- Hard Bone Model: Simulating Type I/II bone quality with high cortical density.
- Normal Bone Model: Representing Type II/III bone quality with moderate cortical and trabecular components.
- Soft Bone Model: Mimicking Type III/IV bone quality with reduced cortical thickness and lower trabecular density.

Each model was constructed using polyurethane foam blocks with density specifications matching the mechanical properties of their respective bone types. Polyurethane bone models (Sawbones; Pacific Research Laboratories Inc., Washington, DC, USA) were used to simulate cancellous bone, and the size of the artificial bone block was 130 mm × 90 mm × 40 mm. Three different types of polyurethane bone models were compared: one with a uniform density of 15 PCF (0.24 g/cm^3^, soft bone), the second one with a uniform density of 30 PCF (0.48 g/cm^3^, normal bone) and the third one with a uniform density of 40 PCF (0.64 g/cm^3^, hard bone). Standard implant fixtures (4.0 mm diameter × 10 mm length, IS II active, Neobiotech Co., Ltd., Seoul, Republic of Korea) were placed in each model using standardized surgical protocols to ensure consistent implant–model interface conditions.

### 2.3. Measurement Devices

**Figure 1:**
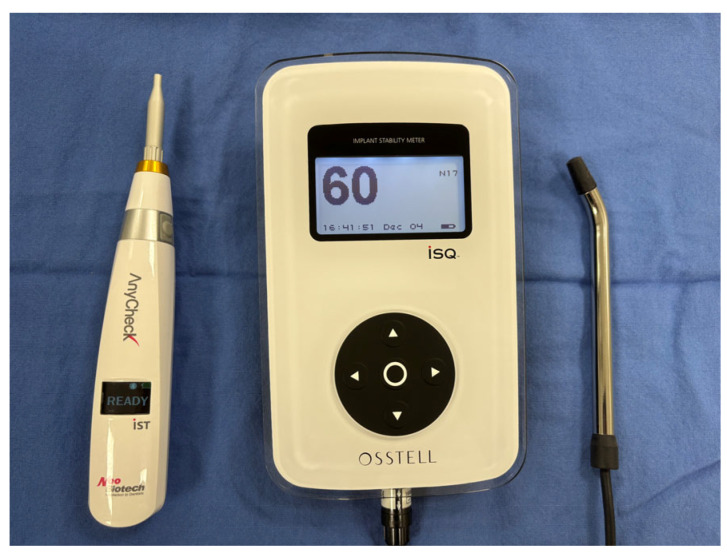
Devices used for implant stability assessment. The AnyCheck IST (Neobiotech, Seoul, Republic of Korea) is shown on the left, and the Osstell ISQ Mentor 2 (Integration Diagnostics AB, Gothenburg, Sweden) is shown on the right.

Osstell ISQ mentor 2 (Integration Diagnostics AB, Gothenburg, Sweden) (Figure 1): The resonance frequency analysis was performed using the Osstell ISQ device with appropriate SmartPeg transducers (Integration Diagnostics AB, Gothenburg, Sweden). Measurements were taken in two perpendicular directions (buccal–lingual and mesial–distal) and averaged for analysis.

Anycheck IST (Neobiotech, Seoul, Republic of Korea) (Figure 1): The Anycheck IST device performs measurements based on damping capacity analysis by applying a controlled percussive force perpendicular to the implant axis. Measurements were obtained by six consecutive taps within approximately 3 s, following the manufacturer’s standardized protocol. Care was taken to maintain consistent angulation and contact pressure across all measurements.

### 2.4. Examiners

Seven dental hygienists participated as examiners in this study, representing a range of clinical experience levels:

- 2 examiners with <2 years of experience.
- 3 examiners with 2–5 years of experience.
- 2 examiners with >5 years of experience.

All examiners received standardized training on both measurement devices prior to data collection, including hands-on practice sessions to ensure familiarity with the measurement protocols.

### 2.5. Measurement Protocol

Each examiner performed measurements 20 times on each bone quality model using both devices in a randomized sequence to minimize systematic bias. The measurement protocol included:

- Device calibration according to manufacturer specifications.
- Standardized positioning with consistent angulation and contact pressure.
- Triplicate measurements for each implant site with each device.
- Blinded data recording to prevent bias between measurements.
- Rest periods between measurements to prevent examiner fatigue.

### 2.6. Statistical Analysis

Statistical analysis was performed using SPSS software (version 28.0). Intraclass correlation coefficients (ICC) with 95% confidence intervals were calculated for both intra-examiner and inter-examiner reliability. Pearson correlation coefficients were calculated to assess the relationship between IST and ISQ measurements. One-way ANOVA was used to compare measurement values across bone quality types and examiner experience levels. Statistical significance was set at *p* < 0.05 for all analyses.

## 3. Results

### 3.1. Overall Reliability

Both the Anycheck IST and Osstell ISQ demonstrated excellent reliability across all measurement conditions. The overall inter-examiner reliability showed ICC values of 0.94 (95% CI: 0.91–0.97) for IST and 0.96 (95% CI: 0.94–0.98) for ISQ, indicating excellent agreement between examiners for both devices. Intra-examiner reliability was consistently high, with mean ICC values of 0.97 ± 0.02 for IST and 0.98 ± 0.01 for ISQ across all examiners and bone quality types.

### 3.2. Inter-Examiner Reliability Across Bone Types

**Figure 2:**
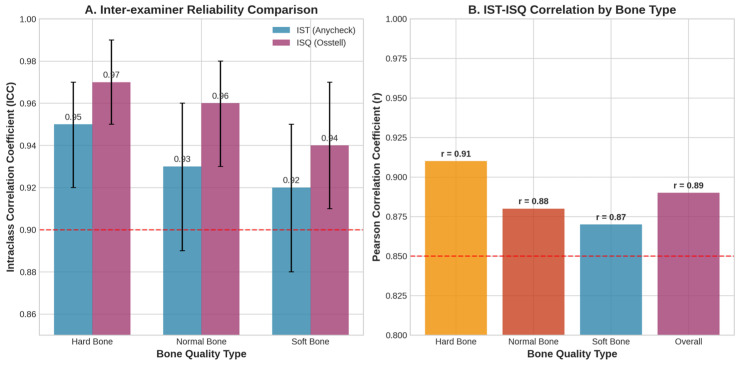
(**A**) Inter-examiner reliability comparison showing excellent ICC values (>0.90) for both IST and ISQ across all bone quality types. Error bars represent 95% confidence intervals. (**B**) Strong positive correlations between IST and ISQ measurements across different bone quality types, with overall correlation r = 0.89.

Table 1 presents the detailed inter-examiner reliability results across different bone quality types. Inter-examiner reliability remained consistently excellent across all bone quality types for both devices (Figure 2).

### 3.3. Correlation Between IST and ISQ

Strong positive correlations were observed between IST and ISQ measurements across all experimental conditions:

- Overall correlation: r = 0.89 (*p* < 0.001).
- Hard bone: r = 0.91 (*p* < 0.001).
- Normal bone: r = 0.88 (*p* < 0.001).
- Soft bone: r = 0.87 (*p* < 0.001).

**Figure 3:**
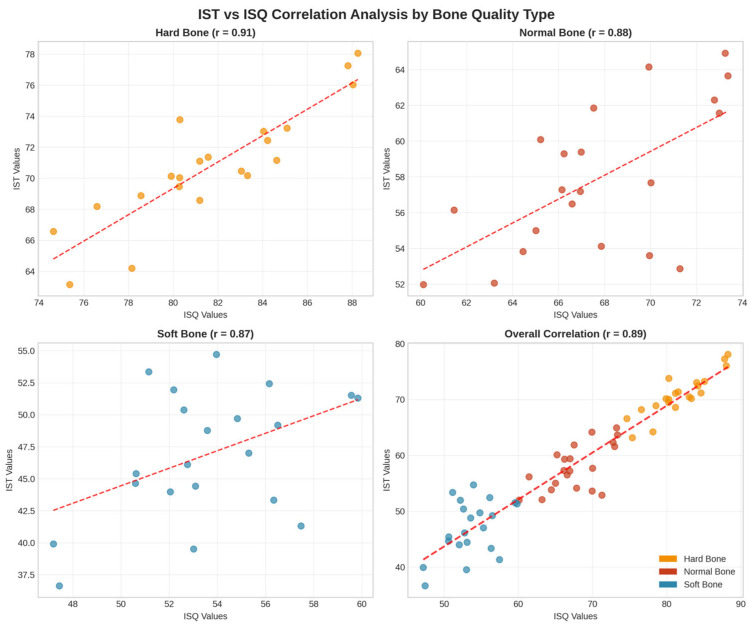
Scatter plot analysis showing strong positive correlations between IST and ISQ measurements across different bone quality types. Individual correlations: Hard bone r = 0.91, Normal bone r = 0.88, Soft bone r = 0.87, with overall correlation r = 0.89. Dashed lines represent linear regression fits demonstrating predictable relationships between the two measurement methods.

The scatter plot analysis (Figure 3) demonstrated linear relationships between IST and ISQ values across all bone quality types, with consistent correlation patterns and minimal outliers. The correlation remained consistently strong regardless of bone quality type, indicating that both devices capture similar aspects of implant stability across different bone densities. The linear regression analysis showed good fit across all bone types, supporting the reliability of both measurement methods.

### 3.4. Bone Quality Effects

**Figure 4:**
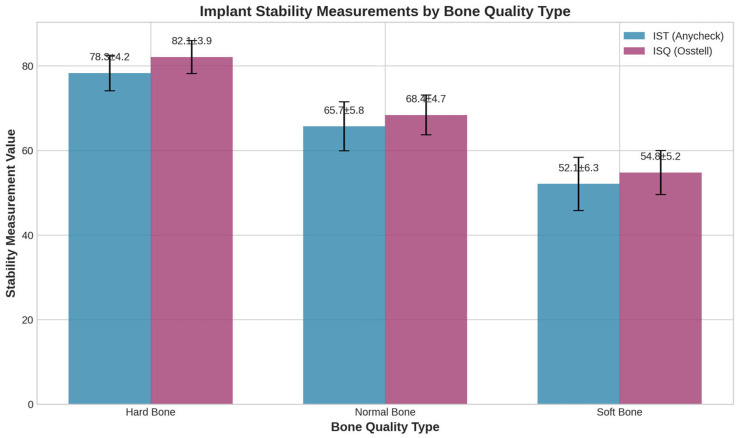
Implant stability measurements by bone quality type. Both IST and ISQ successfully differentiated between hard, normal, and soft bone qualities with statistically significant differences (*p* < 0.001). Values are presented as mean ± standard deviation.

Both devices successfully differentiated between bone quality types, with measurements following expected patterns (Figure 4). ANOVA revealed significant differences between all bone quality groups for both devices (*p* < 0.001), with post hoc analysis confirming significant pairwise differences between all groups.

IST Values (mean ± SD):

- Hard bone: 78.3 ± 4.2.
- Normal bone: 65.7 ± 5.8.
- Soft bone: 52.1 ± 6.3.

ISQ Values (mean ± SD):

- Hard bone: 82.1 ± 3.9.
- Normal bone: 68.4 ± 4.7.
- Soft bone: 54.8 ± 5.2.

### 3.5. Effect of Examiner Experience

**Figure 5:**
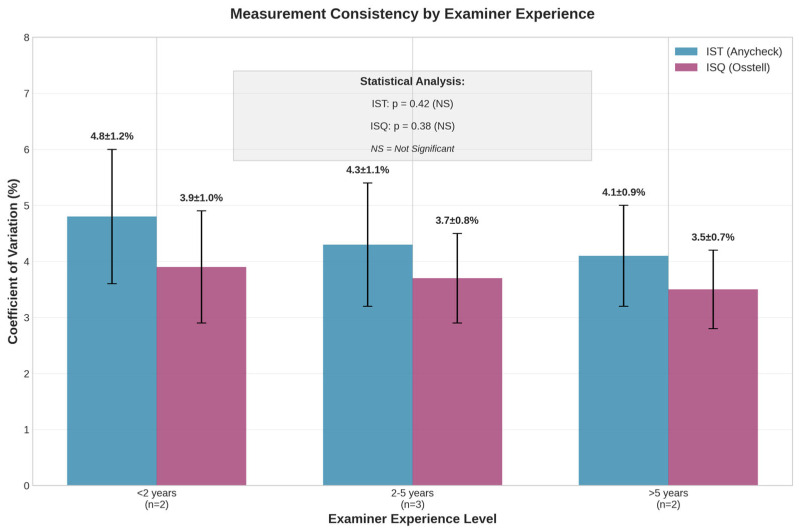
Measurement consistency by examiner experience level. No significant differences were observed between experience groups for either device (IST: *p* = 0.42, ISQ: *p* = 0.38), demonstrating accessibility for practitioners with varying experience levels. Values represent coefficient of variation (%) ± standard deviation.

Analysis of measurements by examiner experience level revealed no significant differences in measurement accuracy or consistency (Figure 5). ANOVA revealed no significant differences between experience groups for either device (IST: *p* = 0.42, ISQ: *p* = 0.38), indicating that both devices can be used reliably by practitioners regardless of their experience level.

Mean Coefficient of Variation by Experience Level:

IST Measurements:

- <2 years’ experience: 4.8% ± 1.2%.
- 2–5 years’ experience: 4.3% ± 1.1%.
- 5 years’ experience: 4.1% ± 0.9%.

ISQ Measurements:

- <2 years’ experience: 3.9% ± 1.0%.
- 2–5 years’ experience: 3.7% ± 0.8%.
- 5 years’ experience: 3.5% ± 0.7%.

### 3.6. Measurement Consistency

The standard error of measurement (SEM) was calculated to assess the precision of both devices:

- IST SEM: 2.1 units (95% CI: 1.8–2.4).
- ISQ SEM: 1.8 units (95% CI: 1.5–2.1).

Both devices demonstrated acceptable measurement precision within the experimental context, with narrow confidence intervals indicating consistent performance across all experimental conditions.

## 4. Discussion

### 4.1. Principal Findings

This study demonstrates that the Anycheck IST provides reliability and inter-examiner agreement comparable to the well-established Osstell ISQ system for implant stability assessment. The excellent inter-examiner reliability (ICC > 0.90) observed for both devices across all bone quality types supports the clinical utility of IST as a reliable alternative to traditional ISQ measurements.

The strong positive correlations (r > 0.85) between IST and ISQ measurements across different bone densities indicate that both devices capture similar underlying mechanical properties of the implant–bone interface. This finding is consistent with previous research by Lee et al. [4], who reported a robust positive correlation (r = 0.981, *p* < 0.01) between ISQ and IST values. These results align with previous biomechanical and clinical reviews suggesting that resonance frequency analysis and damping-based stability assessments reflect similar mechanical characteristics of the implant–bone complex, and that agreement between different non-invasive methods is expected when measurements are interpreted within the same stiffness-based framework [12,13].

### 4.2. Practical Implications and Procedural Considerations

The comparable reliability of IST and ISQ has important clinical implications, particularly considering the practical advantages offered by the IST system. Unlike the Osstell ISQ, which requires SmartPeg attachments and their removal for measurement, the Anycheck IST can perform measurements directly on healing abutments without requiring their removal [3,14]. This feature is clinically significant as it helps prevent accelerated bone resorption that can occur with repeated abutment manipulation, supporting the “one abutment-one time” concept for controlling bone resorption [15].

Lee et al. [4] demonstrated that the diameter of healing abutments (ranging from 4.0 to 6.0 mm) had no statistically significant effect on IST values across different insertion torque values, indicating the device’s versatility and reliability across various clinical scenarios. The improved measurement process offered by the Anycheck device, which applies less force and requires fewer taps (6 times within 3 s compared to Periotest’s 16 impacts), associates it with reduced procedural force and fewer measurement steps while maintaining measurement accuracy [4,5].

### 4.3. Bone Quality Considerations

Both devices successfully differentiated between different bone quality types, with measurements following expected patterns where higher bone density corresponded to higher stability values. This finding aligns with recent research by Lee et al. [10], who demonstrated that both RFA and DCA methods showed positive correlations between primary implant stability and bone density across all implant lengths.

The maintenance of strong correlations between IST and ISQ across all bone types suggests that both methods are similarly sensitive to changes in bone quality and implant–bone interface characteristics. Research by Okuhama et al. [16] revealed that AnyCheck could be effectively used even with provisional crowns, despite the presence of titanium bases that might interfere with percussion response, suggesting the device’s robust measurement capability across various clinical scenarios.

### 4.4. Inter-Examiner Reliability and Accessibility

The finding that examiner experience level did not significantly affect measurement consistency for either device is particularly relevant for clinical implementation. Lee et al. [10] specifically noted that the Anycheck device offers greater ease of use compared to the Osstell system, particularly regarding the measurement process, which makes it more accessible regardless of the observer’s expertise.

The contact-based measurement method of Anycheck simplifies the measurement process compared to the Osstell system, which requires SmartPeg tightening and consistent force application while maintaining constant distance [10]. This accessibility advantage is crucial for widespread adoption of objective stability measurement techniques.

### 4.5. Cost-Effectiveness and Clinical Implementation

The practical advantages of the IST system extend to economic considerations that may influence clinical adoption. The elimination of SmartPeg requirements reduces both initial investment costs and ongoing operational expenses. SmartPegs are implant-system specific consumables that require inventory management and replacement, whereas the Anycheck system can be used across different implant systems without additional components [4,5]. Moreover, the IST system facilitates minimally invasive clinical workflows by enabling implant stability assessment directly through the healing abutment, without the need for abutment removal or replacement for SmartPeg attachment. This reduction in procedural steps not only streamlines clinical protocols but also minimizes patient discomfort and lowers the risk of soft tissue irritation or secondary infection. Clinical studies have validated that IST measurements obtained via damping capacity closely correlate with those from ISQ resonance frequency analysis, supporting reliable and safe monitoring with less peri-implant tissue manipulation. These attributes further enhance practical clinical implementation and may improve procedural efficiency, strengthening the case for widespread adoption of the Anycheck system across diverse implant platforms [2,17,18].

### 4.6. Study Limitations

Several limitations should be acknowledged. The study was conducted using in vitro models rather than clinical patients, which may not capture all aspects of the clinical measurement environment. The study focused on primary stability measurements and did not evaluate the devices’ performance in monitoring stability changes over time. The standardized implant size and placement protocol may not reflect the variability encountered in clinical practice. This study focused exclusively on primary implant stability measured immediately after placement. Secondary stability, which reflects biological osseointegration over time, was not evaluated. Future longitudinal studies incorporating secondary stability measurements are warranted to further validate the clinical applicability of the IST system.

### 4.7. Clinical Decision-Making Framework

Based on the accumulated evidence from this in vitro study, a conceptual decision-making framework can be proposed:

Choose IST (Anycheck) when:

- Minimizing abutment manipulation is prioritized.
- Cost-effectiveness is a primary concern.
- Simplified measurement protocols are preferred.
- Multi-user accessibility is important.

Choose ISQ (Osstell) when:

- Non-contact measurement is preferred.
- Established reference values are prioritized.
- Integration with existing RFA-based protocols is required.

Both systems are appropriate when:

- Objective stability assessment is the primary goal.
- High measurement reliability is required.
- Bone quality differentiation is needed.

## 5. Conclusions

This comparative in vitro study demonstrated that the Anycheck IST provides reliability and inter-examiner agreement comparable to the established Osstell ISQ system for implant stability assessment under standardized experimental conditions. The principal findings of this study can be summarized as follows:

Both IST and ISQ demonstrated excellent inter-examiner reliability across all tested bone quality models, with ICC values exceeding 0.90. Strong positive correlations were observed between IST and ISQ measurements regardless of bone density, indicating that both devices capture similar mechanical characteristics of the implant–bone interface. In addition, both systems consistently differentiated between hard, normal, and soft bone quality models, following expected stability patterns. Examiner experience did not significantly influence measurement consistency for either device, suggesting reliable performance across users with varying levels of clinical experience. Measurement precision for both systems remained within clinically acceptable ranges under the experimental conditions.

Within the limitations of this in vitro model, the IST system demonstrated practical advantages, including the elimination of SmartPeg requirements, reduced procedural steps, and simplified measurement protocols. These features may offer potential benefits in terms of procedural efficiency and operational convenience when compared with conventional resonance frequency analysis systems.

However, it is important to emphasize that this study was conducted using standardized polyurethane bone models rather than real bone tissue. Therefore, the findings should be interpreted strictly within the context of an in vitro experimental design. The results indicate comparable measurement reliability and agreement between IST and ISQ under controlled conditions, but they do not directly establish clinical performance or patient-related outcomes. Further well-designed clinical and longitudinal studies are required to confirm the applicability of these findings in real clinical settings and to evaluate implant stability behavior over time.

## Figures and Tables

**Table 1:** Inter-examiner Reliability Results by Bone Quality Type.

Bone Type	Device	ICC	95% CI	SEM	CV (%)
Hard	IST	0.95	0.92–0.97	1.8	4.2
Hard	ISQ	0.97	0.95–0.99	1.5	3.8
Normal	IST	0.93	0.89–0.96	2.1	4.8
Normal	ISQ	0.96	0.93–0.98	1.7	3.9
Soft	IST	0.92	0.88–0.95	2.4	5.1
Soft	ISQ	0.94	0.91–0.97	2.0	4.3

ICC: Intraclass Correlation Coefficient; CI: Confidence Interval; SEM: Standard Error of Measurement; CV: Coefficient of Variation. No significant differences were found in inter-examiner reliability between bone quality types for either device (*p* > 0.05).

## Data Availability

Data can only be provided or accessed from the corresponding author upon reasonable request.

## Abbreviations

The following abbreviations are used in this manuscript:

IST	Implant Stability Test
ISQ ICC	Implant Stability Quotient Intraclass Correlation Coefficient
